# Facial Paralysis Associated With Lyme Neuroborreliosis in Pediatric Patients: A Multicenter Retrospective Study in Eastern France From 2014-2020

**DOI:** 10.7759/cureus.104205

**Published:** 2026-02-24

**Authors:** Amine Kaake

**Affiliations:** 1 Pediatric Neurology, Assistance Publique-Hôpitaux de Paris (AP-HP) Robert Debre, Paris, FRA

**Keywords:** facial nerve paralysis, lyme borreliosis, lyme neuroborreliosis, lyme's disease, nervous system lyme disease, pediatric emergency department

## Abstract

Introduction: Lyme neuroborreliosis (LNB) is a recognized cause of peripheral facial paralysis (PFP) in children, particularly in endemic regions. Differentiating LNB from idiopathic facial paralysis at initial presentation remains challenging, as early biological confirmation may be delayed. This study aimed to determine the proportion of LNB among children presenting with facial paralysis at two tertiary pediatric centers in Eastern France, to identify clinical, epidemiological, and biological characteristics associated with LNB, and to evaluate local diagnostic and therapeutic practices in order to propose an updated management algorithm.

Methods: We conducted a multicenter retrospective study including patients under 18 years of age presenting with facial paralysis at the pediatric hospitals of Nancy (January 2014 to December 2018) and Reims (January 2014 to December 2020), France. Patients were identified using ICD-10 codes and classified according to final diagnosis. Demographic, epidemiological, clinical, biological, and therapeutic data were extracted from electronic medical records. Comparative analyses were performed to identify characteristics associated with LNB and to evaluate diagnostic and treatment practices.

Results: Among 159 children with facial paralysis, 18 (11.3%) were diagnosed with LNB. Clinical features more frequently observed in LNB included general symptoms (44.4% vs. 15.6%, p<0.05) and associated neurological signs (44.4% vs. 22%, p<0.05). Tick bite history and erythema migrans were infrequently reported. Biological findings such as positive Borrelia serology and cerebrospinal fluid lymphocytic pleocytosis were observed in patients with LNB and contributed to diagnostic confirmation. Complete recovery rates did not differ significantly between groups.

Conclusion: In this endemic region, LNB accounted for a substantial proportion of pediatric facial paralysis cases. Early clinical features, particularly general and neurological symptoms, may help guide diagnostic evaluation before biological confirmation. These findings support the development of a structured management algorithm adapted to endemic settings to improve diagnostic accuracy and optimize patient care.

## Introduction

Peripheral facial paralysis (PFP) in children is characterized by motor deficit of the hemiface, with an annual incidence estimated at 2.7/100,000 in children under 10 and 10.1/100,000 in older children [1]. Diagnosis is clinical, and severity is assessed using the House-Brackmann classification [2,3]. Prognosis is usually favorable, with complete recovery in 85% of idiopathic cases, though infectious causes such as Ramsay Hunt syndrome have poorer outcomes [3-5]. Corticosteroid use in children remains debated, as studies show no clear benefit compared to symptomatic management alone [5-7]. Antivirals have not demonstrated efficacy alone but may accelerate recovery when combined with corticosteroids in severe cases [8-11].

Lyme borreliosis (LB), transmitted by *Ixodes* ticks, is the most frequent vector-borne infection in Europe and North America, with increasing incidence in France [12,13]. Pediatric hospitalizations for LB show neurological involvement in more than half of cases, especially facial palsy [13]. Diagnosis relies on serology and cerebrospinal fluid (CSF) analysis, though early tests may be negative [14,15]. Current French guidelines recommend antibiotics systematically in suspected neuroborreliosis, with favorable outcomes in most cases [16,17].

In endemic regions such as Eastern France, differentiating idiopathic PFP from Lyme neuroborreliosis (LNB) is a major clinical challenge. Epidemiological and clinical predictors such as tick exposure, erythema migrans, general or neurological symptoms, and CSF lymphocytic meningitis can guide diagnosis and management [18,19]. Early identification is crucial to avoid both underdiagnosis, risking long-term sequelae, and overtreatment with unnecessary antibiotics.

This study aimed to determine the proportion of LNB among children presenting with facial paralysis at two tertiary pediatric hospitals in Eastern France (Nancy, 2014-2018; Reims, 2014-2020), to identify clinical, epidemiological, and biological characteristics associated with LNB, and to evaluate diagnostic and therapeutic practices in order to propose an updated management algorithm adapted to endemic settings.

## Materials and methods

Study population

We conducted a retrospective multicenter study including all patients under 18 years of age presenting with facial paralysis to the pediatric emergency departments or hospitalized in pediatric units at the University Hospital of Reims (January 2014 to December 2020) and the Regional University Hospital of Nancy (January 2014 to December 2018). Patients were identified using ICD-10 diagnostic codes G51.0 (facial nerve disorders) and A69.2 (Lyme disease), retrieved from the Medical Information Departments of both centers. Medical records were reviewed to confirm eligibility.

PFP was defined as an acute onset of unilateral or bilateral weakness of the facial muscles consistent with lower motor neuron involvement, as documented by the treating physician. Patients were excluded if facial paralysis was attributed to central neurological causes, tumors, trauma, surgical complications, or other clearly identified non-infectious etiologies. LNB was defined based on the combination of facial paralysis and biological evidence of *Borrelia* infection, including positive *Borrelia* serology in blood and/or cerebrospinal fluid, together with cerebrospinal fluid lymphocytic pleocytosis when lumbar puncture was performed, in accordance with national diagnostic recommendations.

Data collection

Data were extracted retrospectively from electronic medical records (DxCare software, Dedalus Healthcare) using a standardized data collection form. 

Collected Variables

The following is a presentation of the variables involved in the data collection.

Demographic variables: age at presentation and sex.

Epidemiological variables: season of presentation, history of tick bite, and presence of erythema migrans.

Clinical variables: side of facial paralysis (unilateral or bilateral), severity when documented according to the House-Brackmann classification, presence of general symptoms (fever, asthenia), and associated neurological signs (headache, meningeal signs, or other neurological symptoms).

Biological variables: inflammatory markers, *Borrelia *serology in blood, CSF cytology including lymphocyte count, *Borrelia* serology in CSF, and CSF antibody index when available.

Diagnostic and therapeutic management variables: performance and timing of lumbar puncture, specialist consultations (neurology, ENT), imaging studies, antibiotic therapy, corticosteroid therapy, treatment type, dosage, and duration.

Outcome variables: recovery status and time to recovery. Complete recovery was defined as full resolution of facial motor deficit as documented in follow-up clinical examinations. Time to recovery was defined as the interval between initial diagnosis and documented complete recovery.

Follow-up data were obtained from outpatient consultation records when available. Cases without documented follow-up were classified as missing for outcome analysis and excluded from recovery comparisons. Data extraction was performed by reviewing electronic medical records. When information was missing or unavailable, the variable was recorded as missing and excluded from the corresponding analysis. No imputation of missing data was performed.

Practice evaluation

**Figure 1:**
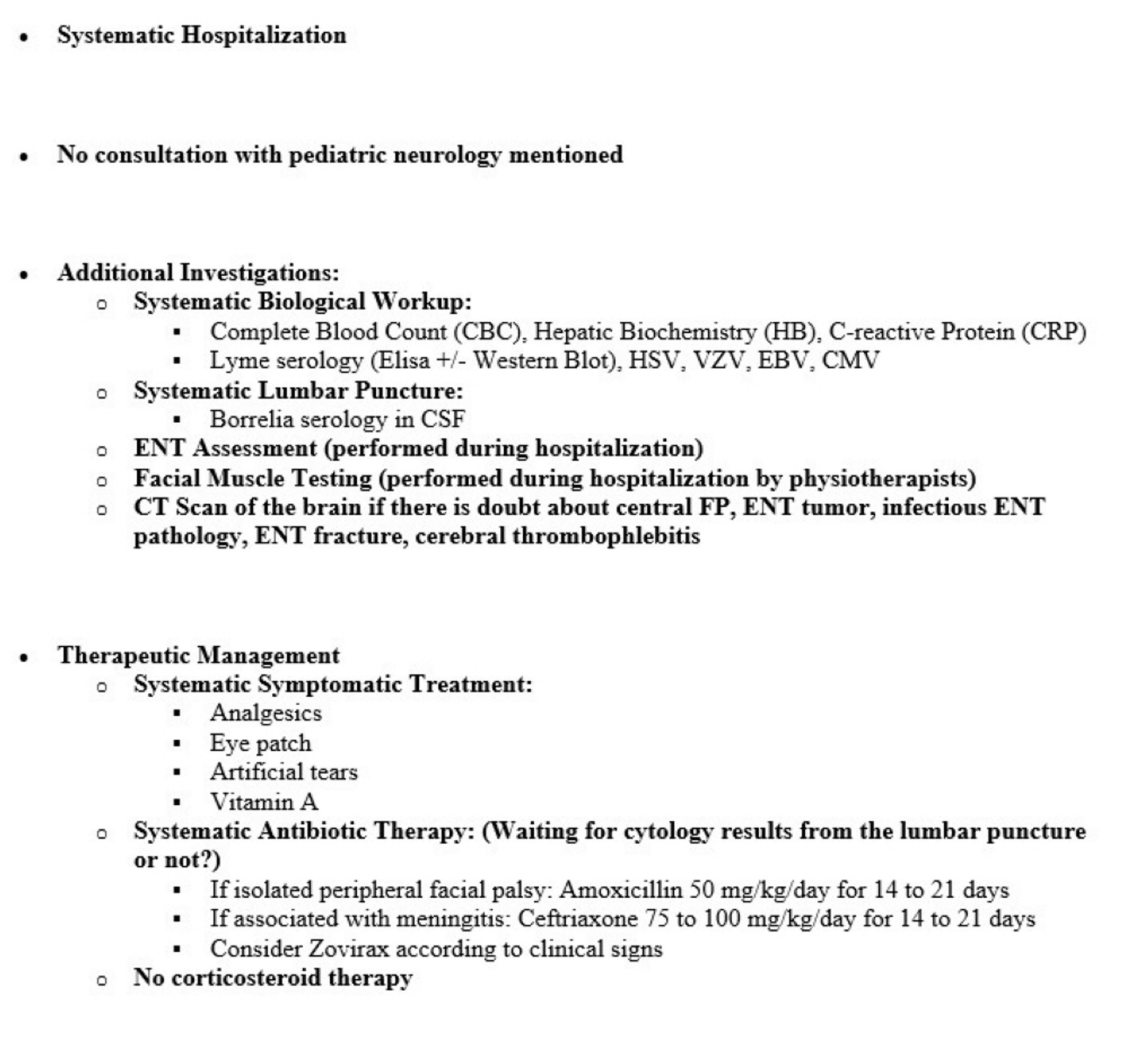
Previous Protocol for the Management of Facial Paralysis in the Pediatric Emergency Department of Nancy, France (before 2019). HSV: Herpes Simplex Virus; VZV: Varicella-Zoster Virus; EBV: Epstein–Barr Virus; CMV: Cytomegalovirus; FP: Facial Palsy; ENT: Ear, Nose, and Throat.

To evaluate diagnostic and therapeutic practices, we analyzed the investigations and treatments performed in both centers and compared them with existing recommendations. In addition, the formal management protocol implemented in the pediatric emergency department of Nancy prior to 2019 was reviewed and used as a reference for evaluating adherence to recommended diagnostic and therapeutic steps (Figure 1). Practice evaluation focused on the performance and timing of biological investigations, lumbar puncture, specialist consultations, and treatment initiation.

Statistical analysis

Qualitative variables were expressed as frequencies and percentages. Quantitative variables were expressed as mean and standard deviation or median and interquartile range, depending on distribution. Comparisons between groups were performed using the Chi-square test or Fisher’s exact test for categorical variables. Recovery time was analyzed as a categorical variable (0-3 months vs >3 months) due to incomplete follow-up data. A p-value <0.05 was considered statistically significant. Analyses were performed using Excel Stats software. Analyses were conducted on available data, and missing values were excluded from the corresponding analyses.

Ethical statement

This retrospective study was conducted in accordance with institutional and national ethical standards. The use of anonymized patient data complied with French regulations governing retrospective clinical research. According to national regulations, formal informed consent was not required for retrospective analysis of anonymized data. No patient or legal guardian expressed opposition to the use of medical data for research purposes.

## Results

**Figure 2:**
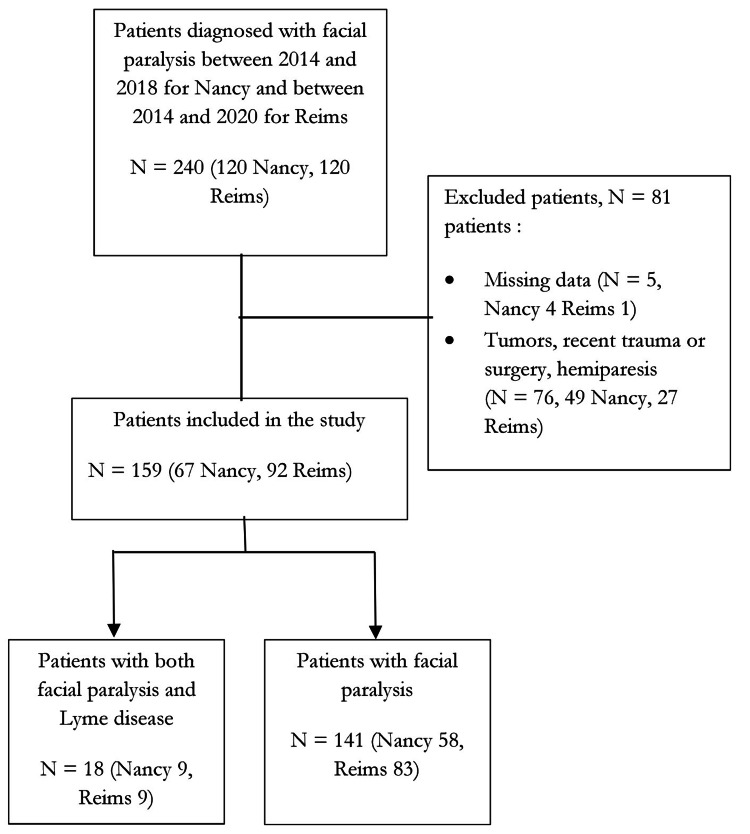
Organizational Chart of the Studied Population in the Two Centers.

A total of 240 patients with a diagnosis of facial paralysis were initially identified through ICD-10 coding at the two participating centers (Nancy: n=120; Reims: n=120). After medical record review, 81 patients were excluded due to inaccessible records, incorrect diagnosis, central neurological causes, traumatic or iatrogenic facial paralysis, or other identified etiologies. The final study population consisted of 159 patients presenting with PFP, including 67 patients from Nancy and 92 from Reims (Figure 2)

Among these patients, 18 (11.3%) were diagnosed with LNB based on clinical presentation and biological findings, while 141 (88.7%) were classified as non-Lyme facial paralysis. Among non-Lyme facial paralysis (n=141), 58 were from Nancy and 83 from Reims, consistent with the total distribution of included patients. The proportion of LNB among facial paralysis cases was 13.4% (9/67) in Nancy and 9.8% (9/92) in Reims.

**Figure 3:**
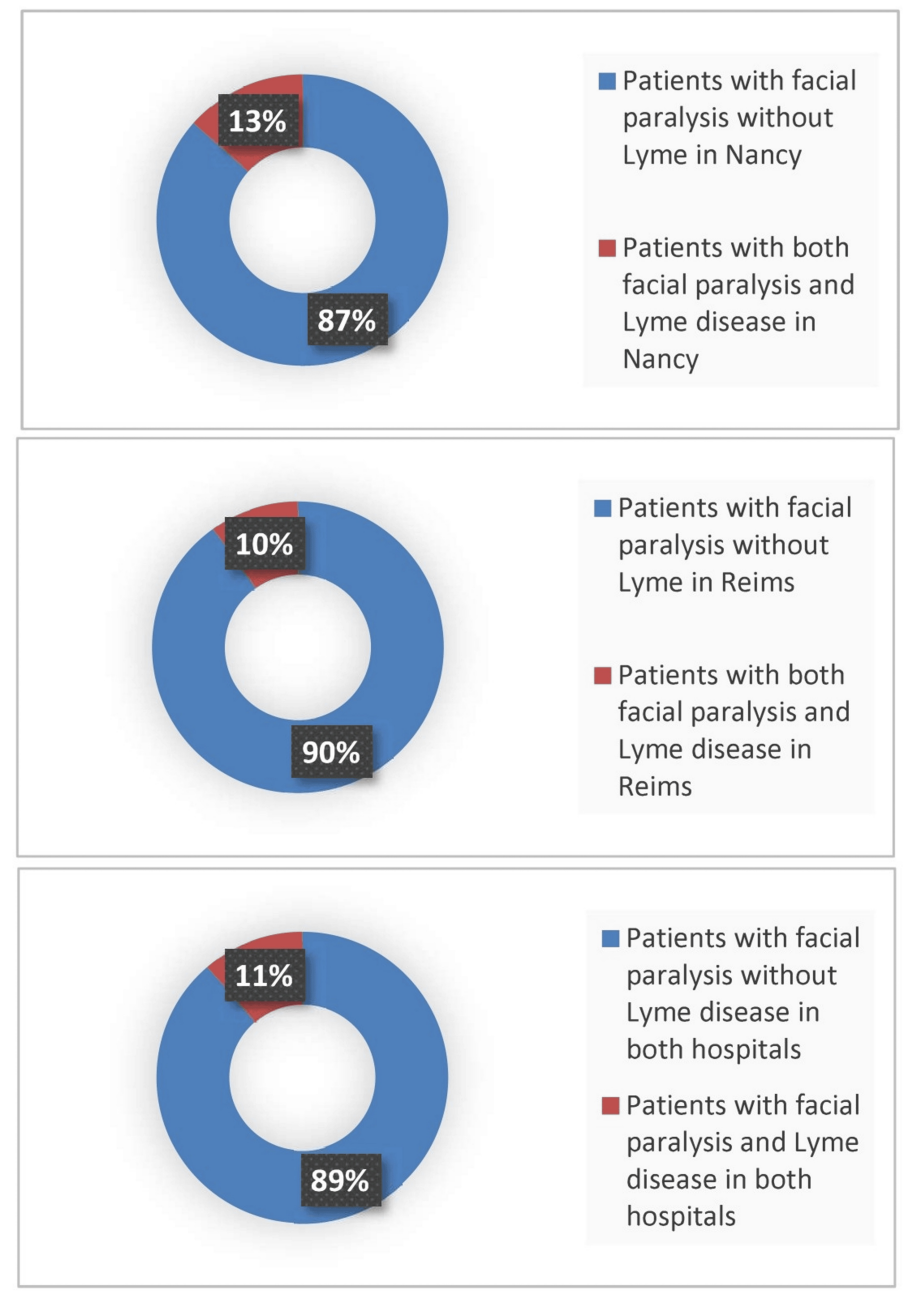
Percentage of Lyme Neuroborreliosis Among Facial Paralysis Cases in Children in Reims and Nancy From 2014 to 2020.

We confirmed the diagnosis of LNB based on the presence of facial paralysis, positive BL serology in serum, and evidence of lymphocytic meningitis. From 2014 to 2018, for Nancy and up to 2020 for Reims, we recorded 159 cases of facial paralysis in children across the two hospitals, with 67 cases in Nancy and 92 in Reims. Among the patients in Nancy presenting facial paralysis, 9 out of 67 cases (13%) were diagnosed as LNB while in Reims, 9 out of 92 cases (10%) were diagnosed as LNB (Figure 3).

We compared demographic, epidemiological, clinical, and biological characteristics between patients diagnosed with LNB and those with non-Lyme facial paralysis (Table 1). No significant differences were observed regarding sex distribution between groups. Age distribution differed slightly between groups (p=0.047), with LNB cases occurring more frequently in children aged 1-12 years.

**Table 1:** Demographic, Epidemiological, Anamnestic, Clinical, Biological Factors and Evolution of Lyme Neuroborreliosis. * A statistically significant difference is observed according to the Pearson chi-square tests or Fisher’s exact test for qualitative variables and the Student's t-test for quantitative variables. ** Diagnostic biological variables (*Borrelia* serology and cerebrospinal fluid findings) are presented descriptively without statistical comparison, as they are part of the diagnostic confirmation criteria for LNB. † Recovery data were available for 18 patients with LNB and 80 patients without LNB. †† Recovery time was analyzed as a categorical variable due to incomplete follow-up data.

Demographic, Epidemiological. Anamnestic, Clinical, Biological factors and Evolution Data	Patients with Facial Paralysis and Lyme (N=18)		Patients with Facial Paralysis without Lyme (N=141)		χ² (df)	P
	N	%	N	%	χ² value	P value
Sex						
Women	11	61	80	56.7	0.01 (1)	0.92
Men	7	39	61	43.3		
Age (years)					7.95 (3)	0.047
<1	0	0	5	3.5		
1-6	8	44.4	35	24.8		
7-12	9	50	52	36.9		
12-18	1	5.6	49	34.8		
Season (From June to Nov)	13	72	68	48.2	2.78 (1)	0.095
Season (From Nov to Jun)	5	28	73	51.8		
Tick Bite	5	28	4	2.8	14.22 (1)	<0.001*
Erythema migrans	5	28	0	0	31.83 (1)	<0.001*
Clinical Signs						
Unilateral	16	88.9	139	98.6	2.80 (1)	0.094
Bilateral	2	11.1	2	1.4		
Severe	3	16.7	13	9.2	0.33 (1)	0.57
General Signs (fever, asthenia)	8	44.4	22	15.6	6.89 (1)	<0.05*
Associated Neurological Signs	8	44.4	31	22	3.22 (1)	<0.05*
Others Signs	8	44.4	56	39.8	0.017(1)	0.90
Inflammatory Syndrome	1	5.5	14	9.9	0.029(1)	0.87
Diagnostic Confirmation Variables **						
Positive Lyme Blood Serology	16	88.9	2	1.4	-	-
Lymphocytic Meningitis	14	77.8	2	1.4	-	-
Positive Lyme Serology in the Cerebrospinal Fluid	10	55.6	0	0	-	-
Positive Cerebrospinal Fluid Index	3	16.66	0	0	-	
Complete Recovery†	18	100	80	56.7	0.52 (1)	0.47
Recovery Time††						
0-3 months	10	55.5	73	51.7	11.82(1)	<0.001
>3 months	8	44.5	7	4.9		

A history of tick bite and erythema migrans was significantly more frequently reported in patients with LNB (p<0.001). However, these findings were present in a minority of LNB. General symptoms, including fever and asthenia, were more frequently observed in patients with LNB (44.4% vs. 15.6%, p<0.05). Associated neurological symptoms, such as headache or meningeal signs, were also more common in the LNB group (44.4% vs. 22%, p<0.05). Biological findings such as positive *Borrelia* serology and cerebrospinal fluid lymphocytic pleocytosis were used as part of the diagnostic confirmation of LNB and are therefore presented descriptively rather than as independent comparative variables.

Predictive factors and evolution of LNB

There was no significant difference regarding the month of the year during which PFP was diagnosed between groups. Regarding epidemiological data, we can observe that 13 (72% )of the PFP on LNB cases were diagnosed during the high-risk season from June to November, compared to only 68 (48.2%) of the other PFP cases. Significant differences were noted between the two groups concerning the history of tick bites and erythema migrans on the skin (Table 1). In the part of clinical signs, there is no significant difference between the groups regarding the bilateral or unilateral side of facial paralysis. However, a significant difference was observed regarding associated general signs (fever, asthenia) and neurological symptoms (such as meningeal syndrome, headaches). No significant difference was found in other associated signs (arthralgia, myalgia, rash). Biological findings, including positive *Borrelia* serology and cerebrospinal fluid lymphocytic pleocytosis, were used as part of the diagnostic confirmation of LNB and are therefore presented descriptively rather than as independent comparative variables (Table 1).

Regarding the evolution of facial paralysis, we compared recovery between the PFP on LNB group and other PFP groups. No significant difference was found in complete recovery. Recovery time was analyzed categorically due to incomplete follow-up data. A higher proportion of patients with LNB had recovery time greater than three months compared to non-Lyme cases, although interpretation is limited by missing follow-up data.

Follow-up data regarding recovery were available for a subset of patients. Complete recovery data were available for 18 patients with LNB and 80 patients with non-Lyme facial paralysis. Complete recovery was observed in all 18 patients with LNBand in 80 patients with non-Lyme facial paralysis, with no statistically significant difference between groups. The median time to recovery appeared longer in the LNB group compared to non-Lyme cases. However, interpretation of recovery outcomes is limited by missing follow-up data and the small number of LNB cases. We also studied the potential effect of oral corticosteroid therapy on the recovery of other PFP cases, dividing patients into those who received corticosteroids (n=61) and those who did not (n=80). No significant difference was found in the recovery time between these two groups (p=0.80) (Table 1).

Analysis of the diagnostic strategy and treatment 

In terms of diagnosis, regardless of the etiology of PFP, a biological workup to identify an inflammatory syndrome was almost always performed in 152 (95.6%) cases. Blood serology for Borrelia was also always performed in 144 (90.6%) cases. Other recommended blood serologies (HSV, VZV, EBV, CMV) were carried out in 87.4 % of cases. As part of cerebrospinal fluid analysis, the lumbar puncture was performed in 17 (94.4 %) cases for PFP due to LNB and 68 (48.2 %) for others. Regarding additional investigations, 97 patients (61%) underwent at least an ENT assessment and muscle testing. A pediatric neurology consultation was performed in 64 cases (40.3%), most frequently in patients with facial palsy with LNB (12 cases; 66.6%). These consultations were primarily conducted during hospitalization, with 37 cases (57.8%) occurring within 24 hours of admission (Table 2).

**Table 2:** Analysis of the Diagnostic Strategy Implemented for Facial Paralysis and the Treatment Administered. ENT: Ear, Nose, and Throat; MRI: Magnetic Resonance Imaging.

Diagnostic Strategy and Treatment Data	Patients with Facial Paralysis and Lyme (N=18)		Patients with Facial Paralysis without Lyme (N=141)		All Patients (N=159)	
	N	%	N	%	N	%
Biological Test	18	100	134	95	152	95.6
Lyme Serology	18	100	126	89.3	144	90.6
Others Serology	18	100	121	85.8	139	87.4
Lumbar Puncture	17	94.4	68	48.2	85	53.5
<24h	9	50	40	58.8	49	57.6
>24h	8	50	28	41.2	36	42.4
ENT Consultation	8	44.4	89	63.1	97	61.0
MRI or Scan	5	27.8	55	39	60	37.7
Neurological Consultation	12	66.6	52	36.8	64	40.3
<24h	3	25	24	46.1	27	42.2
>24h	9	75	28	53.9	37	57.8
Treatment Antibiotics						
Yes	18	100	75	53.2	93	58.5
No	0	0	66	46.8	66	41.5
Immediately	11	61.1	48	64.0	59	63.4
Secondarily	7	38.9	27	36	34	36.6
After Lumbar Puncture or Serology	12	66.6	7	5	19	20.4
Amoxicilline	7	38.9	49	65.3	56	60.2
Third Generation Cephalosporine	11	61.1	7	9.3	18	19.4
Both Drugs	2	11.1	2	1.4	4	4.3
Others Antibiotics	0	0	19	25.3	19	20.4
Recommended Dosage	14	77.8	43	94.6	57	61.3
Duration 21 Days	10	55.5	4	5.4	14	15.05
Other Duration	8	44.5	71	94.6	75	85
Corticosteroids	1	5.5	61	43.3	62	39.0
Immediately	1	5.5	16	26.2	17	27.4
After ENT Consultation	0	0	45	73.7	45	72.6
Prednisolone	1	5.5	57	93.4	58	93.5
Others Steroids	0	0	4	6.5	4	6.5
Recommended Dosage	1	5.5	57	93.4	58	93.5
Duration 10 Days	0	0	19	31.1	19	30.6
Other Duration	1	5.5	42	68.9	43	69.4

Regarding therapeutic management, antibiotic therapy was administered in 18 (100%) of FP with LNB cases, with secondary administration in 7 (38.9%) cases, initiated after the detection of lymphocytic meningitis or serological results. A third-generation cephalosporin (TGC) was initiated immediately in 11 cases (61.1%), while two patients (11.1%) received amoxicillin followed by TGC after cerebrospinal fluid cytology results became available. The recommended dosage was followed in 14 cases (77.8%). Treatment duration was 21 days in 10 patients (55.5%), whereas 8 patients (44.5%) received alternative durations of therapy.

In other PFP cases, antibiotic therapy was administered in 75 patients (53.2%), most often initiated immediately (48 cases; 64%) and primarily with amoxicillin (49 cases; 65.3%). The recommended dosage was adhered to in 43 cases (94.6%). In the non-LNB PFP group, corticosteroid therapy was prescribed in 61 cases (43.3%), mainly as second-line treatment (45 cases; 73.7%) after negative blood serology for Borrelia infection. Prednisone was used exclusively; however, the recommended 10-day treatment duration was followed in only 19 cases (31.1%).

## Discussion

Proportion of facial paralysis associated with LNB

In this multicenter retrospective study conducted in two tertiary pediatric hospitals in Eastern France, we identified 159 cases of PFP among which 18 (11%) were diagnosed with LNB. This proportion is consistent with epidemiological data reported in endemic regions, where LNB represents a significant cause of facial paralysis in children [13]. However, higher proportions have been reported in other endemic regions, particularly in central Europe and in the northeastern and north-central United States, where LNB may account for more than half of pediatric facial paralysis cases. These geographical variations are likely related to differences in *Borrelia* reservoir hosts, tick density, and ecological conditions influencing transmission dynamics [20-22].

Clinical, epidemiological, and biological characteristics associated with LNB

One objective of our study was to identify clinical and biological characteristics associated with LNB in children presenting with facial paralysis in order to improve early diagnostic evaluation in endemic settings.

From an epidemiological perspective, most LNB cases were diagnosed during the high-risk season (June to November), although this difference was not statistically significant compared to non-Lyme facial paralysis cases. Seasonal variation remains an important epidemiological factor to consider, as demonstrated in previous studies conducted at Boston Children's Hospital and in England, which showed that incorporating seasonal risk improves clinical decision-making in pediatric facial paralysis [18,23].

Regarding medical history, tick bite and erythema migrans were significantly more frequently reported in patients with LNB. However, these findings were present in only a minority of cases, which is consistent with previous studies showing that tick bite is reported in 30% to 50% of cases and erythema migrans in only 20% to 30% of patients with neuroborreliosis [13,24-26]. These findings highlight the limited sensitivity of these epidemiological markers and indicate that their absence does not exclude the diagnosis of LNB.

From a clinical perspective, general symptoms such as fever and asthenia, as well as associated neurological symptoms including headache and meningeal signs, were more frequently observed in the LNB group. Similar findings were reported by Fine et al., who demonstrated that children with LNB were more likely to present with systemic and neurological symptoms compared to idiopathic facial paralysis cases [18]. These clinical features may therefore serve as useful indicators to guide early diagnostic evaluation before biological confirmation is available.

Biological findings such as positive *Borrelia* serology in blood and cerebrospinal fluid, as well as lymphocytic pleocytosis, represent key diagnostic confirmation criteria for LNB rather than independent predictive characteristics. These findings are consistent with established diagnostic recommendations and reflect the biological definition of neuroborreliosis [15,25,27]. Their role is primarily confirmatory, particularly when clinical suspicion is present. In endemic regions, the combination of acute PFP and lymphocytic pleocytosis has been shown to have a high predictive value for LNB [28-30].

Importantly, positive blood serology alone does not necessarily confirm active neuroborreliosis, as false-positive results and past infections may occur. Previous studies have shown that blood serology may initially be negative in up to 20% of confirmed neuroborreliosis cases, with subsequent seroconversion occurring later [25,26]. These findings highlight the importance of cerebrospinal fluid analysis and follow-up serological testing in cases with strong clinical suspicion.

Clinical outcomes and recovery

In our study, complete recovery was observed in most patients in both groups, with no statistically significant difference between LNB and non-Lyme facial paralysis cases. However, interpretation of recovery outcomes should be cautious due to missing follow-up data and the relatively small number of LNB cases.

These findings are consistent with previous studies reporting favorable outcomes in pediatric neuroborreliosis when appropriate antibiotic treatment is administered [13,16,24]. Early and appropriate antibiotic therapy is associated with good neurological recovery and low risk of long-term complications.

Regarding corticosteroid therapy, we did not observe a significant difference in recovery time between patients who received corticosteroids and those who did not in the non-Lyme facial paralysis group. These findings are consistent with previous studies showing no clear benefit of corticosteroid therapy in children with idiopathic facial paralysis [5-7]. Current guidelines recommend corticosteroid therapy primarily in cases of severe facial paralysis and when initiated early in the disease course [2].

Evaluation of diagnostic and therapeutic practices

Our study also evaluated diagnostic and therapeutic practices in pediatric facial paralysis. Most patients underwent biological testing, including *Borrelia* serology. However, confirmatory testing using ELISA and Western Blot, as recommended in diagnostic guidelines, was performed in a limited proportion of cases. Follow-up serological testing after initial negative results was also infrequently performed, despite evidence showing delayed seroconversion in some patients [24-26].

Lumbar puncture was performed in most patients with confirmed neuroborreliosis, but cerebrospinal fluid *Borrelia* serology and antibody index were not consistently obtained in all cases. According to current HAS recommendations, cerebrospinal fluid analysis is essential for confirming the diagnosis of neuroborreliosis [16].

Other diagnostic tools, such as PCR testing in cerebrospinal fluid, have limited sensitivity and cannot reliably exclude the diagnosis [28,29]. Emerging biomarkers, such as CXCL13, have shown promising diagnostic performance and may allow earlier detection of neuroborreliosis, although further validation is needed before routine clinical implementation [29,30]. These findings highlight variability in diagnostic practices and emphasize the importance of standardized diagnostic approaches in endemic regions.

Proposed management protocol

**Figure 4:**
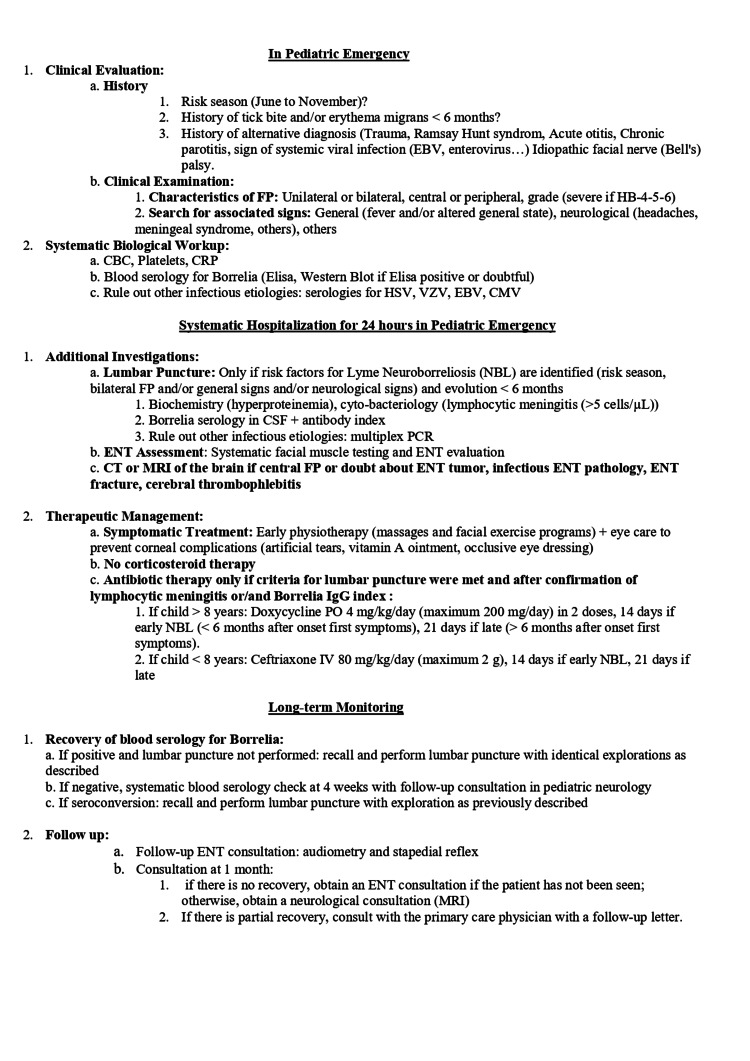
New Protocol for the Management of Facial Paralysis in the Pediatric Emergency. HSV: Herpes Simplex Virus; VZV: Varicella-Zoster Virus; EBV: Epstein–Barr Virus; CMV: Cytomegalovirus; FP: Facial Palsy; ENT: Ear, Nose, and Throat.

Based on our findings and existing literature, we developed a diagnostic and management algorithm for pediatric facial paralysis in emergency settings [13,16,18,23,24]. This proposed protocol integrates clinical assessment, epidemiological context, and appropriate use of biological investigations and lumbar puncture to improve diagnostic accuracy. However, this algorithm is based on retrospective observational data and should be considered as a locally informed clinical framework rather than a validated diagnostic tool. Prospective studies are needed to evaluate its clinical performance and impact on patient management (Figure 4).

Limitations of the study

The data collection for our study relied on ICD-10 coding, with the inclusion of all patients diagnosed with facial palsy (central or peripheral) (G51.0) and/or Lyme disease (A69.2) as the primary or associated diagnosis, as provided by the medical information department. Since there is no specific code for neuroborreliosis, it is likely that this data collection method did not exhaustively include all cases of facial palsy and neuroborreliosis due to coding errors.

This study was retrospective, which made exhaustive data collection impossible, and we lacked a certain amount of information, particularly regarding the progression of facial palsies. Specifically, for 47 cases of non-neuroborreliosis facial palsy, follow-up consultation data was missing. The lack of data on potential recovery has therefore reduced our cohort for this outcome and limited the generalizability of our conclusions regarding therapeutic management and the administration or non-administration of corticosteroids.

This study was also multicentric, conducted in two hospital centers within a known endemic region in France (Grand Est). Our conclusions, particularly concerning incidence and recommended management strategies, cannot be applied to all geographic situations. Another limitation of the study is that the data was collected over different periods, with a longer period in Reims. There is a certain risk of statistical bias with more cases represented in Reims. Furthermore, given that current practices still differ from one hospital to another, it would be interesting to conduct similar research in other regions of France to compare local incidences, practices, and potential progressions, in order to establish a unified management strategy.

## Conclusions

In this retrospective multicenter study conducted in an endemic region, LNB accounted for a substantial proportion of pediatric facial paralysis cases. Clinical features such as general symptoms and associated neurological signs, and the presence of lymphocytic meningitis, may help guide early diagnostic evaluation, although biological investigations remain essential for diagnostic confirmation.

Variability in diagnostic and therapeutic practices highlights the need for structured clinical approaches. The proposed management algorithm may serve as a practical framework to assist clinicians in endemic settings, but further prospective studies are needed to validate its clinical utility. Conducting this same study in other regions of France would allow us to generalize these conclusions and establish a unified management protocol. At the microbiological level, more specific markers such as CXCL13 are currently being evaluated and may need to be incorporated into the assessment of the disease.
